# Spectral Hadamard microscopy with metasurface-based patterned illumination

**DOI:** 10.1515/nanoph-2024-0587

**Published:** 2025-02-07

**Authors:** Yongjae Jo, Hyemi Park, Seho Lee, Inki Kim

**Affiliations:** Department of Biophysics, Institute of Quantum Biophysics, Sungkyunkwan University, Suwon 16419, Republic of Korea; Department of Biophysics, Department of Intelligent Precision Healthcare Convergence, Institute of Quantum Biophysics, Sungkyunkwan University, Suwon 16419, Republic of Korea; Department of Biophysics, Department of Intelligent Precision Healthcare Convergence, Department of MetaBioHealth, Institute of Quantum Biophysics, Sungkyunkwan University, Suwon 16419, Republic of Korea

**Keywords:** Hadamard microscopy, metasurface, hyperspectral imaging, optical sectioning, patterned illumination

## Abstract

Hadamard matrices, composed of mutually orthogonal vectors, are widely used in various applications due to their orthogonality. In optical imaging, Hadamard microscopy has been applied to achieve optical sectioning by separating scattering and background noise from desired signals. This method involves sequential illumination using Hadamard patterns and subsequent image processing. However, it typically requires costly light modulation devices, such as digital micromirror devices (DMDs) or spatial light modulators (SLMs), to generate multiple illumination patterns. In this study, we present spectral Hadamard microscopy based on a holographic matasurface. We noticed that certain patterns repeat within other Hadamard patterns under specific condition, allowing the entire set to be reproduced from a single pattern. This finding suggests that generating a single pattern is sufficient to implement Hadamard microscopy. To demonstrate this, we designed a metasurface to generate an illumination pattern and conducted imaging simulations. Results showed that holographic metasurface-based Hadamard microscopy effectively suppressed scattering signals, resulting in clear fluorescent images. Furthermore, we demonstrated that hyperspectral imaging can be achieved with Hadamard microscopy using dispersive optical elements, as the orthogonality of the Hadamard pattern enables to resolve spectral information. The reconstructed hyperspectral images displayed a color distribution closely matching the synthetic hyperspectral images used as ground truth. Our findings suggest that optical sectioning and hyperspectral imaging can be accomplished without light modulation devices, a capability typically unattainable with standard wide-field microscopes. We showed that sophisticated metasurfaces have the potential to replace and enhance conventional optical components, and we anticipate that this study will contribute to advancements in metasurface-based optical microscopy.

## 1 Introduction

Hadamard matrix is a matrix whose column vectors are mutually orthogonal and consist of elements of +1 and −1 [1], [2]. Numerous methods for constructing Hadamard matrices have been reported, including Sylvester’s [3], [4] and Paley’s [5], [6] construction, making them highly scalable and useful for various applications. Owing to its orthogonality and scalability, the Hadamard basis has been widely used in the field of optical imaging, such as in single-pixel imaging [7], [8], [9], compressed sensing [10], [11], [12], [13], and hyperspectral imaging [14], [15], [16]. Notably, Farhi et al. [17] and Parot et al. [18] proposed and demonstrated the potential of optical sectioning microscopy using the orthogonality of the Hadamard basis. They sequentially illuminated structured light such that each pixel was illuminated with a different Hadamard basis. This allows the separation of the desired signal from the scattered light, resulting in optically sectioned images of the brain tissues. However, the sequential illumination of multiple patterns on a sample requires expensive light modulation devices such as digital micromirror devices (DMDs) or spatial light modulators (SLMs).

In this study, we introduce a holographic metasurface-based Hadamard illumination method to replace conventional light modulating devices. Metasurfaces, composed of nanostructure arrays, are optical components that offer extraordinary optical modulation performance with high efficiency. Due to their small unit-cell pitch, typically smaller than the wavelength (∼*λ*/2), they avoid higher-order diffraction and can achieve high efficiencies (∼96.9 %) for hologram generation [19]. In contrast, SLMs and DMDs, which have larger pixel pitches (>10 μm), suffer from higher-order diffraction, limited reflectivity, and reduced fill factors, resulting in maximum total light efficiencies below ∼70 % [20]. Moreover, SLMs operate at half efficiency under unpolarized illumination, as they only modulate a single polarization direction. Metasurface can also generate holograms with a wide viewing angle (>75°), calculated as $\theta =2\sin^{-1}\left(\frac{\lambda }{2p}\right)$ where *p* and *λ* are the pixel pitch and wavelength, respectively. This wide viewing angle enables large-field illumination, thereby improving imaging speed. In contrast, SLMs and DMDs have much narrower viewing angles (<∼3°).

We found that only one Hadamard pattern is sufficient to reproduce the entire set of required patterns, as patterns made using Paley’s Hadamard matrices exhibit self-similarity (also referred to as periodicity). Hadamard microscopy can thus be implemented using a single illumination pattern with an appropriate shift. To validate this concept, we designed a holographic metasurface and obtained a hologram of the Hadamard pattern using the wave propagation method. Subsequently, we simulated Hadamard microscopy, including illumination and subsequent image processing for optical sectioning, which yielded remarkably clear images with reduced scattering. Compared to previous reports that employed DMD [17], [18], we exhibited same concept can be achieved with a more cost effective and simpler optical setup using metasurfaces.

Furthermore, we demonstrate the potential of hyperspectral imaging with Hadamard microscopy by exploiting dispersive optical components. Typically, imaging techniques that locally activate individual fluorophores, such as photoactivated localization microscopy (PALM) [21], [22], [23], [24], [25], [26], and stochastic optical reconstruction microscopy (STORM) [27], [28], [29], [30], enable hyperspectral imaging using dispersive optics [31], [32], [33], [34]. Because they sparsely activate fluorophores, spectrally dispersed point spread functions (PSFs) do not overlap, allowing for the acquisition of intact spectral information. Hadamard illumination also provides sparsely separated signals after the decoding process, enabling the acquisition of spectral information with minimal crosstalk. As a proof of concept, we conducted imaging simulations of spectral Hadamard microscopy on previously obtained confocal fluorescent images and successfully reconstructed the hyperspectral data.

Consequently, our proposed approach demonstrates the simultaneous feasibility of hyperspectral imaging and optical sectioning. While hyperspectral confocal microscopy achieves both hyperspectral imaging and optical sectioning, it suffers from low dwell time and low signal-to-noise ratio (SNR) due to its point-by-point raster-scanning data acquisition [35]. In contrast, our spectral Hadamard microscopy offers significant advantages in cost and SNR by employing a holographic metasurface for Hadamard illumination to acquire spectral data from multiple positions simultaneously. Despite tradeoffs between spatial and spectral resolution and acquisition time, our imaging simulations highlight the feasibility of spectral Hadamard microscopy without relying on conventional light modulators. This innovative approach paves the way for cost-effective and efficient hyperspectral imaging systems.

## 2 Theory

### 2.1 Hadamard matrix and patterned illumination

Hadamard matrix of order *m*, $H_{m}\in {\left\{-1,1\right\}}^{m\times m}$ , is a matrix whose column vectors are mutually orthogonal consisting of elements of +1 and −1 and satisfying the condition $H_{m}H_{m}^{T}=mI_{m}$ , where *I*_*m*_ is the *m* × *m* identity matrix [1], [2]. Encoding signals using Hadamard basis allows for the decoding of signals, even when they are mixed. In previous reports, Farhi et al. [17] spatially separated unwanted scattering from desired fluorescent signals, achieving background removal via patterned illumination based on Hadamard basis. However, this approach requires expensive electronic devices to create multiple patterns.

**Figure 1:**
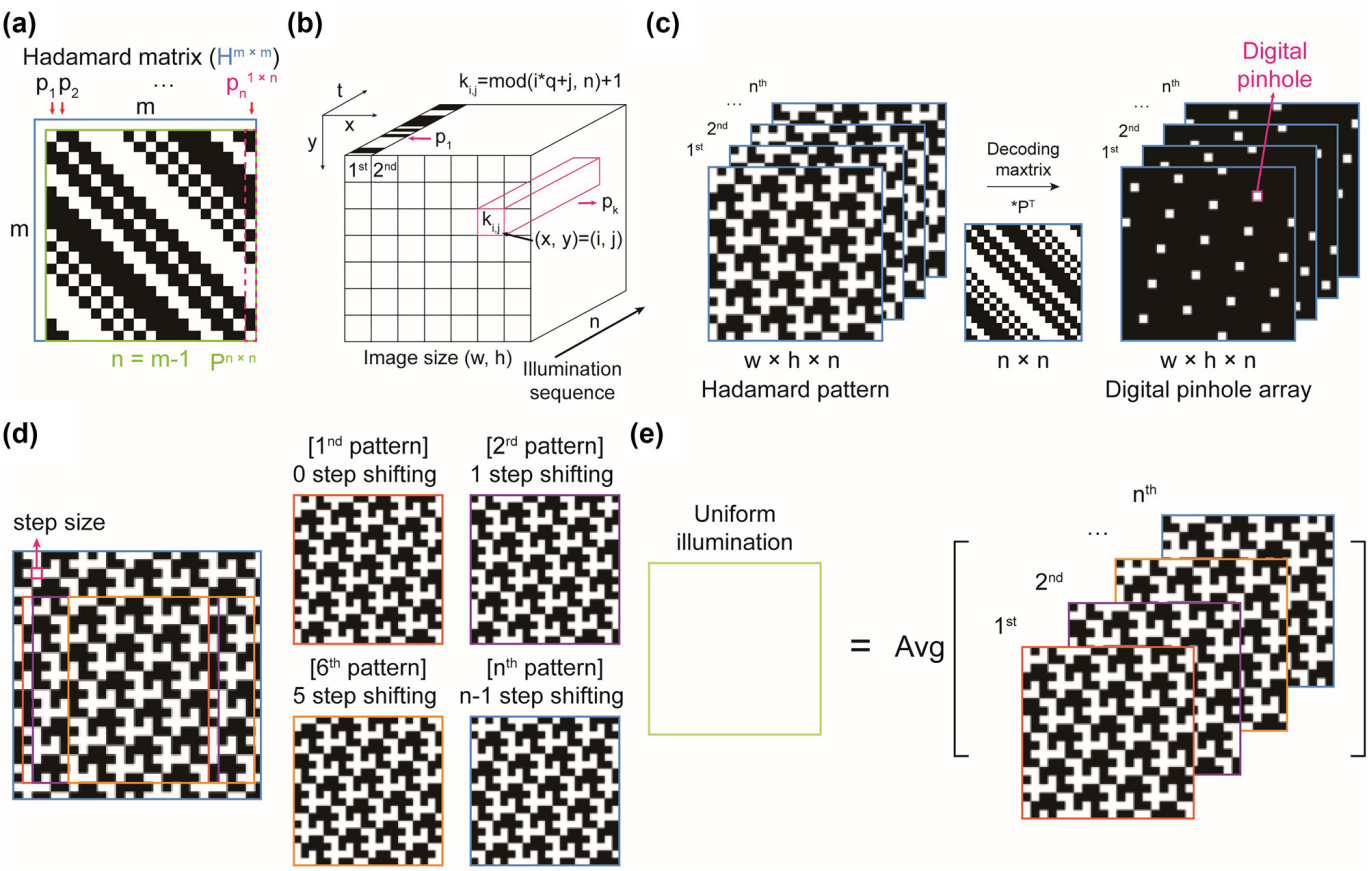
Concept of Hadamard patterns and their self-similarity. (a) Schematic representation of the Hadamard matrix, *H*, and the modified Hadamard matrix, *P*. (b) Arrangement of the Hadamard vector, *p*, for patterned illumination. The number of patterns is determined by the length of the Hadamard vector. We used the parameters $\left(n,q\right)=\left(19,5\right)$ for generating illumination patterns. (c) Decoding process in Hadamard microscopy, which involves the dot products *P* ^*T*^. (d) Self-similarity of the Hadamard matrix, illustrating how the entire set of illumination patterns can be reproduced by shifting a single pattern based on its self-similarity. (e) The average of all Hadamard pattern results in uniform illumination. Wide-field microscopy imaging modality can be achieved by averaging images illuminated by Hadamard patterns.

Among possible Hadamard patterns, some exhibit self-similarity, allowing the entire sequence of patterns to be reproduced from a single pattern. Specifically, Paley’s Hadamard matrices, particularly those constructed using Paley construction I [36], enable the generation of Hadamard patterns with self-similarity due to their circular matrix structure. The conditions for constructing Paley’s matrices are (i) *m* must be a multiple of 4 and (ii) *n* = *m* − 1 must be a prime number. These conditions provide valid *n* values as (3, 7, 11, 19, 23, 31, 43, …). In circular matrices like Paley’s Hadamard matrices, any column vector can be obtained by taking repeated cyclic permutations of another column vector, resulting in self-similarity of Hadamard patterns (Figures S1 and S2). To demonstrate this, we used a Hadamard matrix of order 20 (*H*_*m*_, *m* = 20, *n* = 19), excluding the first row and column as they are 1-vector of size *m* (Figure 1(a)). Since light illumination cannot have negative values – only “on” and “off” states are possible – we modified the matrix as follows:

(1) $$P_{n}=\frac{H_{m}\left[2:n,2:n\right]+1}{2},where\;P_{n}\in {\left\{0,1\right\}}^{n\times n},n=m-1$$

(2) $$P_{n}\cdot P_{n}^{T}=\frac{\left[\left(n+1\right)I_{n}+n-3\right]}{4}\;and\;P_{n}=P_{n}^{T}$$

(3) $$p_{k}\cdot p_{r}=\left\{\begin{array}{c} \frac{n-1}{2}\;if\;k=r\;\\ \frac{n-3}{4}\;if\;k\neq r\; \end{array}\right.\;,\;where\;p\in {\left\{0,1\right\}}^{n}$$

where *p*_*k*_ and *p*_*r*_ represent the *k*th and *r*th column vectors of the matrix *P*_*n*_, respectively. We chose *n* = 19 throughout this study, resulting in *p*_*k*_ ⋅ *p*_*r*_ = 9 for the dot product of a vector with itself $\left(r=k\right)$ , while *p*_*k*_ ⋅ *p*_*r*_ = 4 for the dot product of different column vectors $\left(r\neq k\right)$ . In Hadamard microscopy, each sample plane position is illuminated in the sequence of column vectors *p*, which contain binary elements {0, 1} corresponding to the “on” and “off” states, respectively (Figure 1(b)). The *k*th column vector *p*_*k*_ is assigned to pixels $\left(x,y\right)=\left(i,j\right)$ with $k_{i,j}\in \left\{1,2,\ldots ,n\right\}$ determined as $k_{i,j}=\mathrm{mod}\left(i*q+j,n\right)+1$ , where *q* is a parameter used to maximize spatial separation of identical vectors (Figure 1(b)) [17]. In this study, we adopted *q* = 5 to ensure the digital pinholes were evenly distributed, thereby minimizing signal interferences (Figure S3). Consequently, a total of *n* = 19 patterns, each of size of *w* × *h* were generated for Hadamard illumination. These patterns were transformed into a spatially separated digital pinhole array by taking the dot product of $P_{n}^{T}$ with the obtained image stack, corresponding to the decoding process (Figure 1(c)). The arrangement of digital pinhole arrays depends on the *q* value, which determines the horizontal distance between digital pinholes in adjacent rows. The vertical distance between pinholes in adjacent columns is approximately calculated as $round\left(\frac{n}{q}\right)$ (Figure S3). Selecting an appropriate *q* value is crucial to ensure that the digital pinhole arrays are distributed uniformly and sparsely enough to prevent overlapping of spectral and scattering signals. As a rule of thumb, $q=round\left(\sqrt{n}\right)$ provides a uniformly distributed arrangement, as the vertical and horizontal distances become similar. Deviating from this value results in a biased arrangement of digital pinholes, which increases the risk of overlapping of spectral and scattering signals.

Hadamard illumination offers advantages in illumination time. For *n* = 19, the Hadamard illumination provides a 9-fold increase in exposure time for each pixel compared to direct illumination of the pinhole array pattern over the same period, resulting in reduced noise (Figure 1(c)). Notably, the Hadamard patterns under the condition $\left(n,q\right)=\left(19,5\right)$ exhibit self-similarity, indicating that all *n* patterns can be reproduced from a single pattern through appropriate shifting (Figure 1(d)). This self-similarity enables Hadamard illumination without needing DMDs or SLMs. Averaging all *n* patterns without the decoding process yields uniform illumination, essentially same to standard wide-field illumination (Figure 1(e)).

## 3 Methods

### 3.1 Holographic metasurface design

**Figure 2:**
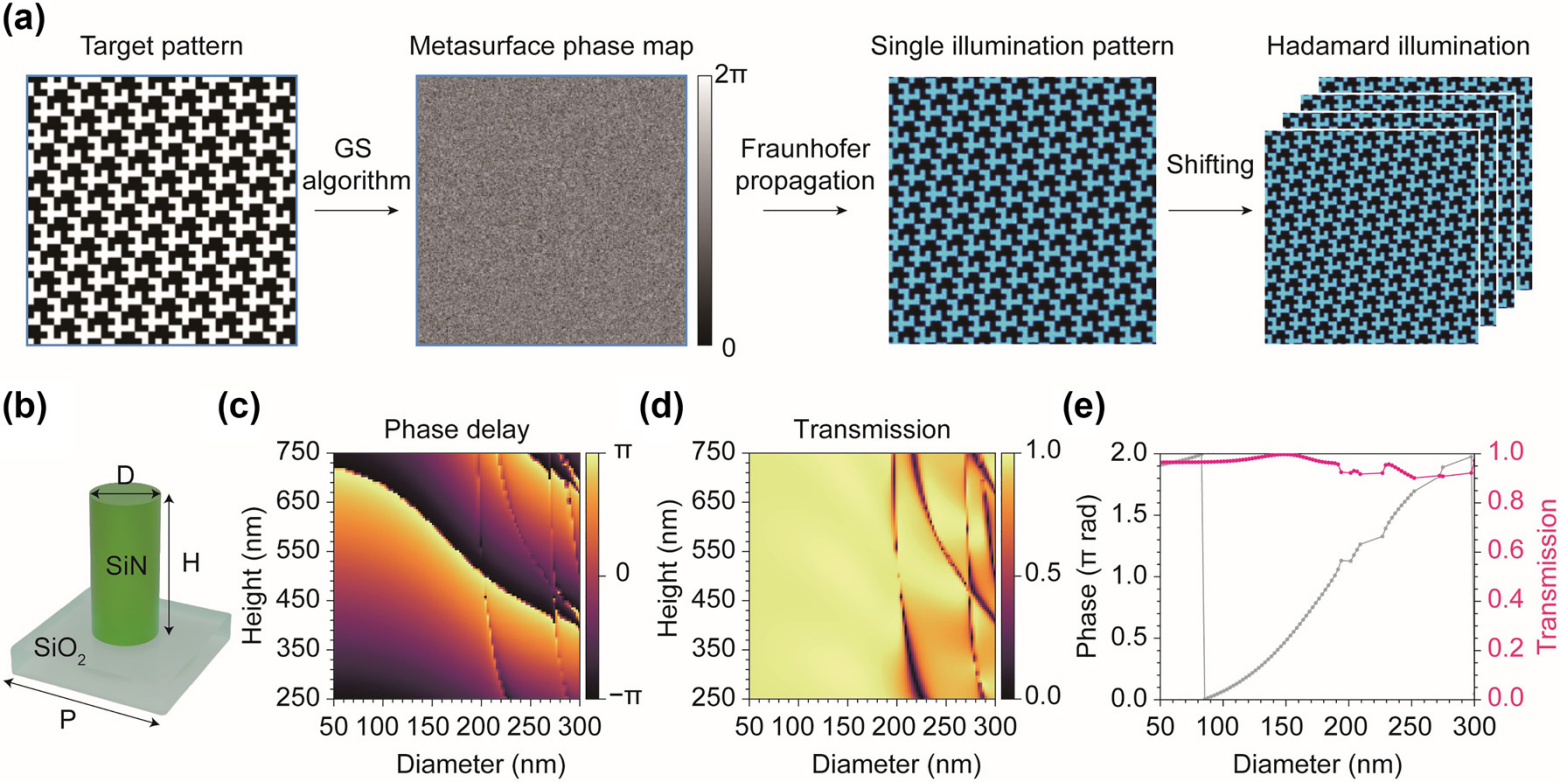
Metasurface design for hologram generation. (a) The metasurface phase map was designed using the GS algorithm with a target Hadamard pattern. A hologram of the Hadamard pattern was obtained through wave propagation simulations. The remaining patterns can be derived by shifting the hologram. To suppress speckle patterns, we averaged 4 shifted images that maintained the same pattern as the original due to self-similarity. This process can be easily reproduced using a motorized stage in a real setup. (b) Structure and parameters of cylindrical meta-atom. D, diameter; H, height; P, period. (c) and (d) Optimization of phase delay (c) and transmission (d) with respect to the diameter and height of the meta-atom. (e) Transmission and phase delay with respect to the diameter at a height of 746 nm. At this height, the meta-atom covers the entire 2π phase with transmission higher than 90 %.

Designing holographic metasurface were performed using numerical simulation except measurements of complex refractive index of silicon nitride (SiN). We designed a metasurface for patterned illumination using the Gerchberg–Saxton (GS) algorithm to generate a hologram of the first Hadamard pattern. For this, we assumed a metasurface diameter of 100 µm and a 330 nm period for a meta-atom arrangement operating at a wavelength of 488 nm. Numerical Fraunhofer propagation was applied to obtain the illumination pattern (Figure 2(a)). For the imaging simulation, the hologram pattern was shifted appropriately to generate additional patterns. In a real imaging system, shifted patterns can easily be achieved using a motorized stage, which is commonly available in conventional optical microscopy. Holography often encounters speckle noise due to the interference of coherent light, which disrupts uniform illumination and introduces artifacts. To address this issue, four identical patterns obtained at different positions were averaged, which can be accomplished by shifting and averaging the hologram in a real system. This compensation is feasible because of the self-similarity of the patterns. The speckle pattern can be further reduced using partially coherent or incoherent light sources [37], [38], [39].

For the meta-atom design, rigorous coupled-wave analysis (RCWA) simulations were performed using TORCWA, a Python library for RCWA [40]. SiN with low attenuation in the visible range was adopted for the cylindrical meta-atoms operating at 488 nm (Figure 2(b)). To obtain the complex refractive index, a SiN film was experimentally fabricated using plasma-enhanced chemical vapor deposition (PECVD, Oxford, PlasmaPro 100 Cobra) on a SiO_2_ substrate. Ellipsometry measurements were then performed, yielding a complex refractive index of 2.101 + 0*i* at 488 nm. To find optimal meta-atoms covering the full 2π phase with high transmission, RCWA simulation was conducted by varying the height (250–750 nm) and diameter (50–300 nm) while keeping the period fixed at 330 nm (Figure 2(b)–(d)). We selected a height of 746 nm, where the meta-atoms cover the full 2π phase delay across the simulated diameter range (Figure 2(e)) and limited the selection to meta-atoms with transmission above 90 %.

### 3.2 Virtual optical setup for imaging simulation

**Figure 3:**
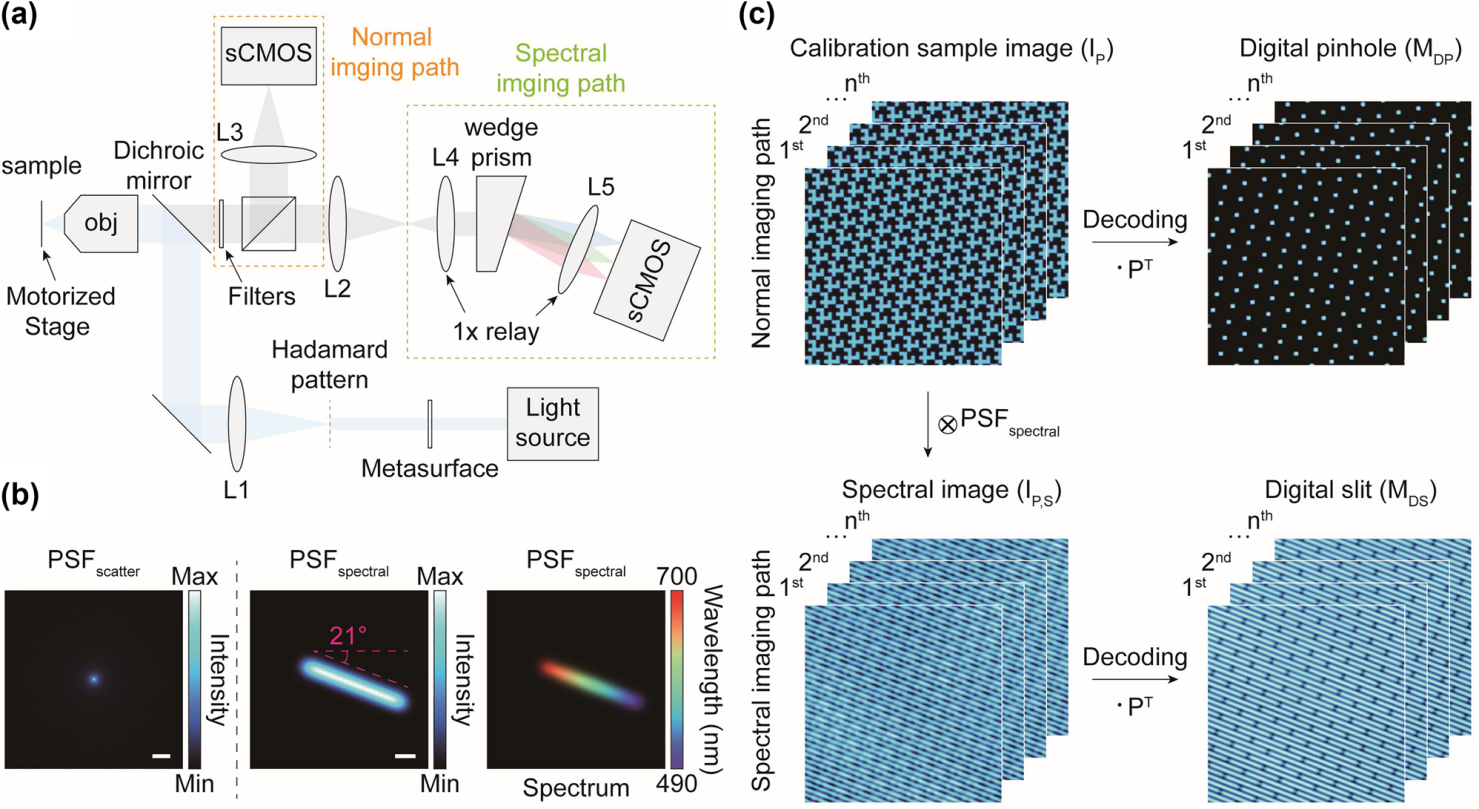
Virtual optical setup and calibration step of the proposed Hadamard microscopy. (a) Virtually designed optical setup for Hadamard microscopy. The normal imaging and spectral imaging paths serve as detection paths for optical sectioning and hyperspectral imaging, respectively. To conduct realistic simulations as close to actual conditions as possible, the virtual setup was designed based on the specifications of commercially available optical components. Additionally, details such as the magnification and NA used in the virtual design were utilized for imaging simulations. (b) Intensity the distribution of the scattering PSF (left), and the intensity *d* (middle) and spectral distributions (right) of the spectral PSF. Scale bars, 10 μm (left) and 5 μm (middle). (c) Calibration of the Hadamard microscope to define the digital pinhole and slit, which are utilized for optical sectioning and hyperspectral imaging, respectively. The circled cross and dot symbols represent convolution and dot product, respectively.

A virtual optical setup was designed to demonstrate the high feasibility of implementation the spectral Hadamard microscope (Figure 3(a)). The setup includes illumination, normal imaging, and spectral imaging paths. All imaging simulations were conducted based on the system parameters of the virtually designed optical setup. In the illumination path, we assumed that a hologram of the Hadamard pattern was generated by illuminating 488 nm laser light onto the metasurface. The hologram is then relayed through the first lens, L1, and the objective lens to the sample plane with a 10×, NA 0.3 objective lens and a relay system that demagnifies the hologram pattern by a factor of 75. In the imaging path, we assumed that a fluorescence signal was collected after passing through the 490 nm long path dichroic mirror and the filter set, including a 490–700 nm bandpass filter and a 488 nm notch filter to block the excitation light. The signal was then split by a beam splitter into two paths: the normal imaging path and the spectral imaging path (Figure 3(a)). Theoretically, fluorescence signals are recorded on the sCMOS camera detector with a pixel size of 6.5 μm and 10× system magnification in the normal imaging path, resulting in an effective pixel size of 0.65 μm. In contrast, spectrally dispersed images are obtained in the spectral imaging path using the prism [31], [32]. In the spectral imaging path, the fluorescence signals are collimated by lens L4, spectrally separated by the wedge prism, and then refocused onto the sCMOS camera by lens L5. The two lenses, L4 and L5, formed a 1× relay system to maintain system magnification, with their focal length selected to ensure that the dispersed signals do not overlap.

## 4 Results and discussion

### 4.1 Calibration

To simulate optical imaging, we defined point spread functions (PSFs), including imaging PSF ( $PSF_{\mathrm{img}}\in R^{w\times h}$ ), scattering PSF ( $PSF_{\mathrm{scatter}}\in R^{w\times h}$ ), and spectral PSF ( $PSF_{\mathrm{spectral}}\in R^{w\times h\times C}$ ), where *w* × *h* represents the image size and *C* is the number of spectral datapoints (Figure 3(b)). The Gaussian function with *σ* = 0.54 pixels was used for *PSF*_*img*_. The *PSF*_*scatter*_ was obtained through a Monte Carlo simulation of light scattering in tissues [41], [42], using parameters that included a tissue scattering coefficient of 100 cm^−1^, excitation and emission wavelengths of 488 nm and 550 nm, respectively, a NA of 0.3, and an imaging depth of 50 μm [43]. These parameters are typically valid for the sectioned tissues. The simulation was accelerated using a GeForce RTX 4090 GPU in a Windows 11 environments.

The *PSF*_*spectral*_ was obtained by slightly shifting *PSF*_*img*_, where the shifting distance proportional to the spectral dispersion. The shift direction was determined based on the arrangement of the digital pinhole arrays, which is governed by the *q* value, to ensure that the *PSF*_*spectral*_ do not overlap each other. Selecting the *q* value involves considering several factors including the thickness of spectral dispersion, spectral resolution, wasted pixels, and overlap of scattering signals to prevent signal interferences while maximizing spectral resolution (Figure S3). If the pinholes are unevenly distributed, the imaging system becomes more susceptible to the spectral signal overlap compared to system with evenly distributed pinholes. Optimal conditions for the maximum dispersion length and angle were determined to be 26 μm (∼40 pixels) and −21.0° rotation, respectively, with a digital pinhole size of 1.95 μm, corresponding to three pixels in the image (Figure 3(b)). This configuration for *PSF*_*spectral*_ maximizes the number of spectral sampling (∼40 channels) without crosstalk for the proposed optical setup. The averaged *PSF*_*spectral*_ over the spectral dimension has an elongated and tilted intensity profile, corresponding to be recorded by the camera in the spectral imaging path (Figure 3(b)). Increasing the digital pinhole size improves the number of spectral sampling but reduces optical sectioning performance.

Calibration is essential for accurately determining the positions of the digital pinholes for optical sectioning and digital slits for hyperspectral imaging (Figure 3(c)). Thin and uniformly distributed fluorescent samples with broad emission spectrum are suitable for this calibration [17], [18]. The fluorescent image of the calibration sample, $I_{P}\left(x,y;k\right)$ , under illumination with the *k*th Hadamard pattern, $I_{H}\left(x,y;k\right)$ , in the normal imaging path can be represented as follows:

(4) $$\begin{array}{c} I_{P}\left(x,y;k\right)=O\left(x,y\right)\times I_{H}^{\prime }\left(x,y;k\right)\\ where\;k\in \left\{1,2,\ldots ,n\right\} \end{array}$$

where $O\left(x,y\right)\in R^{w\times h}$ is the object and, $I_{H}^{\prime }\left(x,y;k\right)=I_{H}\left(x,y;k\right)*PSF_{\mathrm{img}}$ , which is the *k*th Hadamard pattern convolved by *PSF*_*img*_ [44]. The *PSF*_*img*_ can be obtained through both measurements and simulation [45]. In this study, we used a Gaussian function with *σ* = 0.54 pixels to simulate *PSF*_*img*_ [46]. Three-dimensional image data $I_{P}\in R^{w\times h\times n}$ is obtained after sequential illumination with the Hadamard patterns, where the subscript *P* represents the phantom. Consequently, the image *I*_*P*_ simulates the fluorescent images of the calibration phantom with sequential Hadamard illumination. Taking the dot product between *I*_*P*_ and $P_{n}^{T}$ for decoding yields the digital pinhole arrays *M*_*DP*_ (Figure 3(c)):

(5) $$M_{DP}=I_{P}{}^{\circ}P^{T},where\;M_{DP}\in R^{w\times h\times n}$$

where ° denotes the dot product. According to the equation (3), *M*_*DP*_ has maximum and minimum values of 9 and 4 for the condition $\left(n,q\right)=\left(19,5\right)$ , respectively. By subtracting 4 as an offset value, *M*_*DP*_ can be treated as a binary matrix to reject scattering signals while preserving the desired fluorescence signals at the digital pinholes.

Images in the spectral path, $I_{P,S}\in R^{w\times h\times n}$ , can be simulated by convolving the averaged spectral PSF over the spectral dimension, $Avg\left(PSF_{\mathrm{spectral}}\right)\in R^{w\times h}$ , with *I*_*P*_, mimicking the dispersion effect of a wedge prism (Figure 3(c)). The fluorescent image of the calibration sample with spectral dispersion, $I_{P,S}\left(x,y;k\right)$ , under illumination with the *k*th Hadamard pattern in the spectral imaging path can be represented as follows:

(6) $$I_{P,S}\left(x,y;k\right)=I_{P}\left(x,y;k\right)*Avg\left(PSF_{\mathrm{spectral}}\right)$$

The *I*_*P*,*S*_ represents the images of a spectrally dispersed Hadamard pattern. Unfortunately, due to the overlap of dispersed signals, *I*_*P*,*S*_ cannot be directly used to obtain a hyperspectral image. Thus, a decoding process must be applied to *I*_*P*,*S*_ using the Hadamard code $\left(P^{T}\right)$ , similar to the method used for obtaining the digital pinhole. Repeatedly decoding *I*_*P*,*S*_ by taking the dot product with *P* ^*T*^ results in digital slit arrays, *M*_*DS*_.

(7) $$M_{DS}=I_{P,S}{}^{\circ}P^{T},where\;M_{DS}\in R^{w\times h\times n}$$

where *M*_*DS*_ represents the regions for spectral sampling. This step is crucial for defining specific wavelength for each pixel. To assign exact wavelength within digital slit, calibration samples with multiple narrow fluorescent bands are preferred [31]. Unfortunately, the spatial resolution of hyperspectral images is lower than that of optically sectioned images because spectral information can only be acquired at the locations of digital pinholes. Under current conditions, the digital pinholes span three pixels, making the spatial resolution of the hyperspectral image three times lower than that of the optically sectioned image.

There are tradeoffs between acquisition time, imaging field-of-view (FOV), as well as spatial and spectral resolution (Figure S4). For example, demagnifying the Hadamard pattern by a factor of two increases the imaging area and spectral resolution by factors of four and two, respectively, due to the increased inter-pinhole spacing (Figure S4(a)). However, this also reduces the spatial sampling of the hyperspectral image, because each digital pinhole covers four pixels. As a result, the same spectral information acquired at a single digital pinhole must be assigned to four different pixels, thereby lowering the spatial resolution of the hyperspectral image. On the other hand, increasing inter-pinhole distance improves optical sectioning performance by reducing scattering overlaps. Oversampling spectral data between digital pinholes by slightly shifting the Hadamard pattern can interpolate additional spatial information, although it has drawbacks in terms of imaging time. For example, *N* ^2^ times more scanning is required if the digital pinhole size is *N*.

Another strategy to enhance spectral resolution is to use Hadamard patterns with a larger parameter *n*, which increases the spacing between pinholes (Figure S4(b)). Since Hadamard patterns illuminate each pixel $\left(n+1\right)/2$ times – corresponding to the number of “on” states in a length *n* Paley’s vector – the SNR remains unchanged as long as the total acquisition time *t* remains constant. If the total acquisition time for a full Hadamard illumination of length *n* is *t*, then illumination time per frame is *t*/*n*. Because each pixel is illuminated $\left(n+1\right)/2$ times during sequential illumination, the total exposure time per pixel over the entire acquisition is $\left(n+1\right)t/2n$ . Since SNR is proportional to the square root of exposure time [47], it can be expressed as SNR $∼\sqrt{\left(n+1\right)t/2n}$ . Comparing the SNR for different *n* values $\left(n_{1}\text{ and }n_{2}\right)$ at the same total acquisition time *t* gives a ratio $\frac{SNR_{n_{1}}}{SNR_{n_{2}}}∼\sqrt{\frac{1+\frac{1}{n_{1}}}{1+\frac{1}{n_{2}}}}$ , which approaches 1 as *n* increases. This indicates that, for sufficiently large *n* at a fixed total acquisition time *t*, increasing *n* does not significantly affect the SNR. Consequently, increasing *n* can enhance spectral resolution without compromising SNR or acquisition time, provided that pattern shifting and image acquisition are fast. As the illumination time per pattern $\left(t/n\right)$ shortens with larger *n*, a precise and fast motorized stage is required.

### 4.2 Imaging simulation in tissue sample

To demonstrate the feasibility of optical sectioning and hyperspectral imaging, we prepared a 4-channel fluorescent image of a brain organoid using confocal microscopy. The tissue was stained with DAPI and antibodies, including MAP2, Iba1, and GFAP, which are markers for the nucleus, neurons, microglial cells, and astrocytes, respectively. In this study, we generated synthetic hyperspectral data from the 4-channel fluorescent image to simulate hyperspectral imaging. The number of spectral channels was expanded from 4 to *C*, the number of sampled wavelengths, by linearly mixing the ground truth images with known fluorescent spectra. To generate a synthetic hyperspectral image as ground truth, $O_{H}\in R^{w\times h\times C}$ , we computed the dot products of the 4-channel fluorescent image, $I_{R}\in R^{w\times h\times 4}$ , and the emission spectrum data of four fluorescent dyes, $F\in R^{4\times C}$ . This calculation yielded *O*_*H*_ = *I*_*R*_°*F* (Figure S5). Here, *F* represents the matrix of the stacked emission spectra of the four fluorescent dyes. The emission spectra of DAPI and Alexa Fluor 488, 546, and 647 were used. Imaging simulations were categorized into normal and spectral imaging paths.

**Figure 4:**
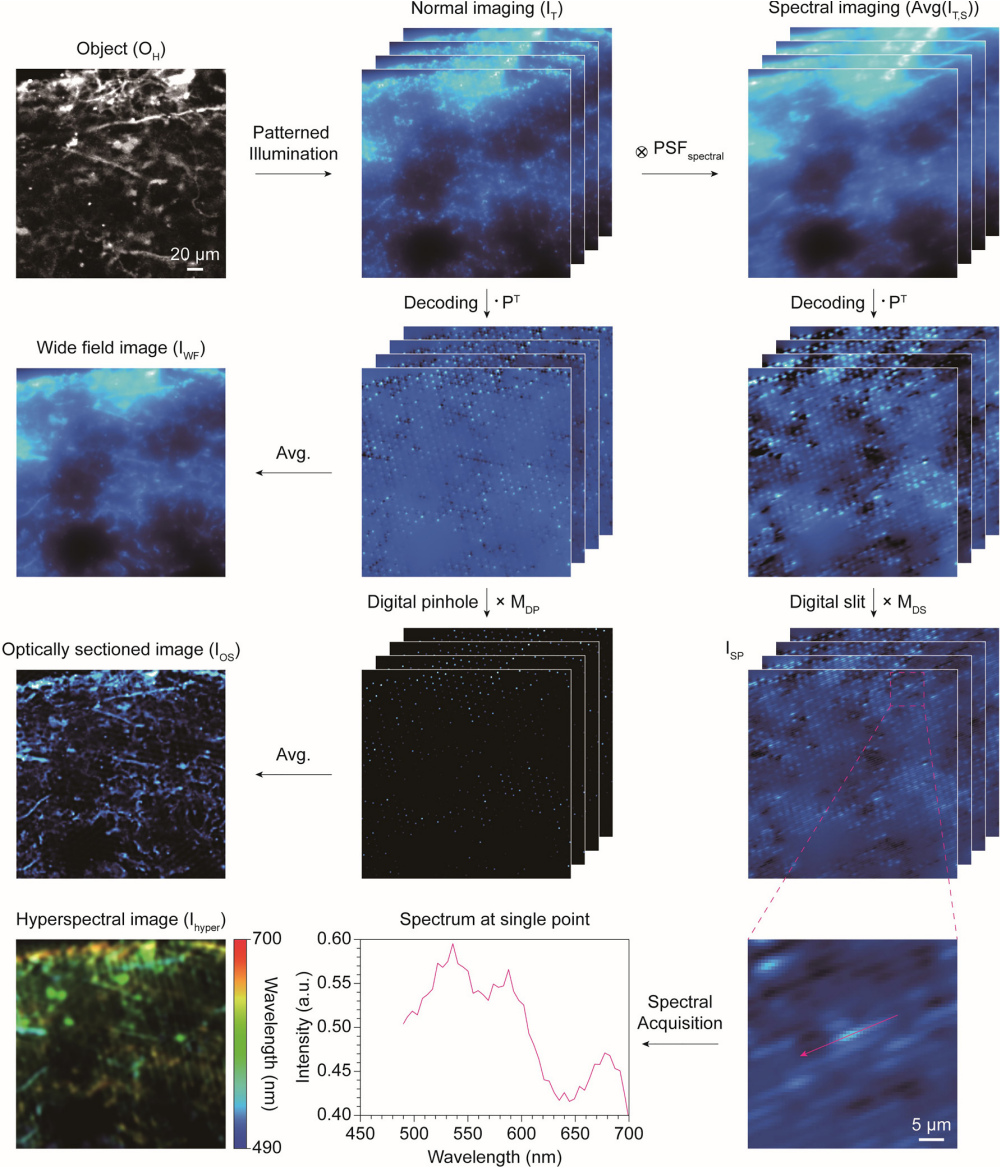
Image processing simulation of metasurface-based Hadamard microscopy. The imaging simulations are classified into optical sectioning and hyperspectral imaging. A 4-channel confocal microscopy image was used to create ground truth. For optical sectioning, we first simulated imaging in a scattering environment under Hadamard illumination. After decoding, a simple average produces a blurred wide-field image, whereas an optically sectioned image is obtained by performing element-wise multiplication with digital pinhole before averaging. In contrast, hyperspectral imaging includes the convolution of the spectral PSF. After decoding and multiplying the digital slit, a spectrally dispersed image is obtained. The hyperspectral image was reconstructed by gathering the spectral information distributed along with the digital slit. The circled cross and dot symbols represent convolution and dot product, respectively.

As a proof of concept, we first performed an imaging simulation in the normal imaging path. Assuming a tissue environment, scattering was accounted for by convolving the *PSF*_*scatter*_ with an object illuminated by Hadamard patterns [42]. The *PSF*_*scatter*_ was obtained through Monte Carlo simulation of photon scattering in scatter media. The ground truth image *O*_*H*_ was averaged over the spectral dimension to simulate a monochrome image recorded in the normal imaging path, where spectral data cannot be distinguished.

(8) $$I_{T}\left(x,y;k\right)=\left[Avg\left(O_{H}\right)\times I_{H}^{\prime }\left(x,y;k\right)\right]*PSF_{\mathrm{scatter}}\left(x,y\right)$$

where $I_{T}\left(x,y;k\right)$ is the tissue image obtained under the *k*th Hadamard pattern illumination. The term in the square bracket in equation (8) represents the simulated grayscale image of an object with hyperspectral information under Hadamard illumination. The convolution with *PSF*_*scatter*_ introduces scattering effect into the simulated image. The resulting images exhibited significant blurring compared with the ground truth due to scattering (Figure 4). This blurring became more evident after decoding $\left(I_{T}{}^{\circ}P^{T}\right)$ . The regularly arranged bright dots represent the desired signals, while the haze surrounding them represents undesired scattering. Simply averaging this image stack produced a normal wide-field image, *I*_*WF*_. The wide-field image was highly blurred due to scattering, which disrupted the resolving of the microstructures of cells. However, by element-wise multiplication of the digital pinhole arrays with the decoded image stack, $\left(I_{T}{}^{\circ}P^{T}\right)\times M_{DP}$ , the scattering around the desired signals was significantly suppressed. Notably, it is crucial to generate an evenly distributed pinhole array with an appropriate *q* value to prevent overlap of scattering signals. Overlapping scattering signals significantly reduces optical sectioning performance as the scattering signals near the center of the pinholes cannot be filtered out (Figure S3). Averaging this image stack yielded an optically sectioned image, $I_{OS}=Avg\left(\left(I_{T}{}^{\circ}P^{T}\right)\times M_{DP}\right)$ , without significant background scattering, similar to confocal microscope images. Periodic grid artifacts inevitably appeared between the digital pinholes due to smooth edges, and a Gaussian filter was applied to suppress these artifacts.

Simulating hyperspectral imaging in the spectral imaging path is more complex than in the normal imaging path due to the inclusion of the spectral dimension [48], [49]. Incorporating the spectral dimension, the spectral image, $I_{T,S}\in R^{w\times h\times n\times C}$ , was expressed as follows:

(9) $$\begin{array}{ll} I_{T,S}\left(x,y;k,c\right) & =\left[\left(O_{H}\left(x,y;c\right)\times I_{H}^{\prime }\left(x,y;k\right)\right)*PSF_{\mathrm{spectral}}\right.\\ & \;\times \left.\left(x,y;c\right)\right]*PSF_{\mathrm{scatter}}\left(x,y\right) \end{array}$$

where $c\in \left\{1,2,\ldots ,C\right\}$ represents a specific spectral channel index. It is worth noting that convolving *PSF*_*spectral*_ for all *C* spectral channels should be performed for all *n* Hadamard illumination patterns. Averaging *I*_*T*,*S*_ over the spectral dimension results in spectrally dispersed images recorded by a monochrome camera, represented as $Avg\left(I_{T,S}\right)\in R^{w\times h\times n}$ . The scattering and spectral dispersion hinder the identification of structural features in these images (Figure 4). However, as in the normal imaging path, decoding and element-wise multiplication with the digital slit, represented as $I_{SP}=\left(Avg\left(I_{T,S}\right){}^{\circ}P^{T}\right)\times M_{DS}$ , resulted in a more refined spectral dispersion with highly suppressed scattering noise. Regularly aligned lines contain spectral information that spread across multiple pixels along the direction of the digital slit (*I*_*SP*_ in Figure 4). Hyperspectral data for each pixel can be obtained by measuring intensity profiles along these lines.

Although *I*_*SP*_ contains spatially distributed spectral information, it must be reassigned to spectral dimensions for hyperspectral image reconstruction (Figure 4). To achieve this, *I*_*SP*_ was rotated by 21.0°, corresponding to the tilt angle of *PSF*_*spectral*_ (Figure 3b), to align the spectral dispersions horizontally. Rearranging the spectral dispersion horizontally, rather than keeping it in the diagonal direction, facilitates easier reassignment of spectral data at the spectral dimension. Then, digital pinhole arrays *M*_*DP*_ were used as a weighting function to extract the spectrum at specific pixels where digital pinholes were located, as the spectral signals originated from these points. Consequently, the reconstructed hyperspectral image, $I_{\mathrm{hyper}}\in R^{w\times h\times C}$ , can be obtained as follows (Figure 4):

(10) $$\begin{array}{ll} I_{\mathrm{hyper,k}}\left[x_{o},y_{o},1:C;k\right] & =M_{DP}\left[x_{o},y_{o};k\right]\times R\left(I_{SP}\right)\\ & \;\times \left[x_{o}:x_{o}+\left(C-1\right),y_{o};k\right] \end{array}$$

(11) $$I_{\mathrm{hyper}}\left[x_{o},y_{o},1:C\right]=\overset{n}{\underset{k=1}{\sum }}I_{\mathrm{hyper,k}}\left[x_{o},y_{o},1:C;k\right]$$

where $R\left(I_{SP}\right)$ represents the rotated *I*_*SP*_, *I*_*hyper,k*_ is the *k*th hyperspectral image under *k*th Hadamard illumination pattern, and *C* is the number of spectral channels. The available number of spectral channels (*C*) is determined by the digital pinhole configuration and the parameter *n* (Figures S3 and S4). Equation (10) indicates that the intensity distribution from position $\left(x_{o},y_{o}\right)$ to $\left(x_{o}+\left(C-1\right),y_{o}\right)$ , corresponding to the spatially dispersed spectrum originating from $\left(x_{o},y_{o}\right)$ , is assigned to spectral channels ranging from $I_{\mathrm{hyper}}\left[x_{o},y_{o},1\right]$ to $I_{\mathrm{hyper}}\left[x_{o},y_{o},C\right]$ . It is worth noting that data acquisition is performed only along the *x*-axis, as the spectral dispersion is rotated horizontally (Equation (10)). Alternatively, the spectral dispersion can be rotated vertically, allowing spectral data to be acquired along the *y*-axis. Importantly, the weighting function $M_{DP}\left[x_{o},y_{o}\right]$ prevents unwanted signals from pixels where digital pinholes do not exist, thus *M*_*DP*_ = 0. A Gaussian filter was also applied to attenuate periodic artifacts.

**Figure 5:**
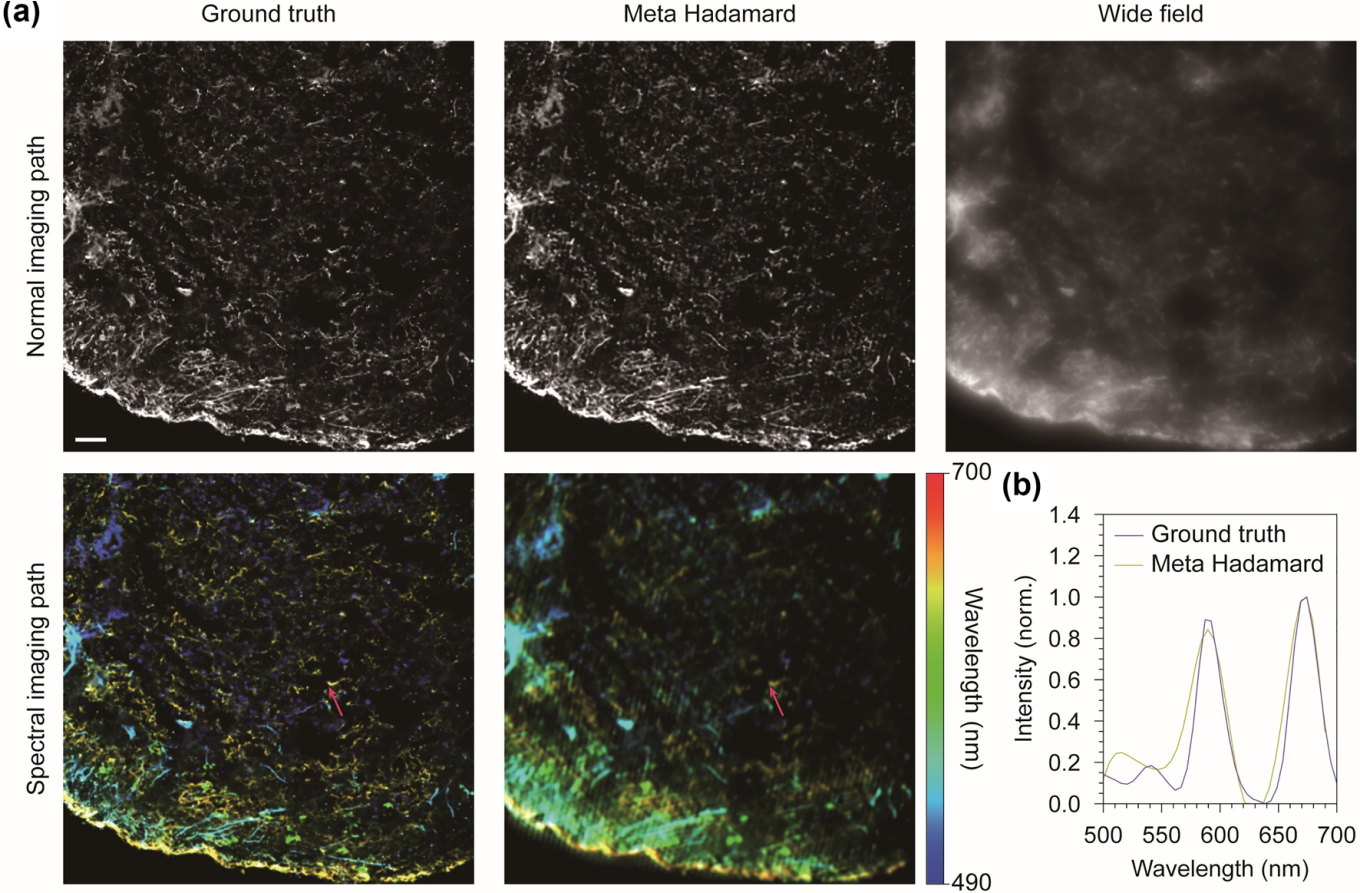
Comparison of the simulation results with ground truth. (a) The ground truth was compared with the imaging simulation results of metasurface-based Hadamard microscopy. The results show that the optical sectioning achieved by the proposed method is comparable to the ground truth, while the wide-field image is highly blurred due to scattering. Although the hyperspectral image exhibits lower spatial resolution compared to the ground truth, the color distribution closely matched that of the ground truth hyperspectral image. The magenta arrows in the hyperspectral images indicate the spectral sampling point. Scale bar, 50 μm. (b) Comparison of the spectral data acquired at the sampling point (indicated by the magenta arrows in (a)) between the ground truth and the spectral Hadamard microscopy.

Finally, we compared our simulation results with ground truth and simulated wide-field images. As previously mentioned, ground truth images were generated using 4-channel confocal microscopy images. The 4-channel image was averaged over the color channels to create a monochrome image. The emission spectra of four dyes, including DAPI, Alexa Fluor 488, 546, and 647, were used to generate synthetic hyperspectral images (Figure 5(a)). Since the confocal microscope effectively rejects background scattering, the ground truth of normal and hyperspectral images displays clear and distinct structural features. Notably, the proposed metasurface-based Hadamard microscope demonstrated optical sectioning performance comparable to the ground truth, whereas the cellular structures in the wide-field image were barely distinguishable due to scattering. These comparisons highlight the optical sectioning capabilities of the proposed technique. Although the hyperspectral image reconstructed using the proposed method showed slightly lower spatial resolution than the ground truth, its spectral distribution closely matched that of the ground truth (Figure 5(b)). Reducing the digital pinhole size improves spatial resolution but compromises spectral resolution. Optical sectioning performance in the hyperspectral image was also slightly lower than in normal optical sectioning, as the digital slit could not completely remove blurring along its direction. Nonetheless, this work demonstrates the feasibility of hyperspectral imaging, which is not achievable using a wide-field microscope. Notably, this advancement can be realized without the need for costly electronic devices for optical modulation.

## 5 Conclusions

In conclusion, we have demonstrated the implementation of a hyperspectral Hadamard microscope based on a holographic metasurface. By leveraging self-similarity, we found that a single pattern was sufficient to reproduce a full set of Hadamard patterns, enabling Hadamard microscopy without requiring electronic devices for light modulation. The necessary Hadamard pattern was generated through numerical simulation of the optical propagation of the holographic metasurface. As a proof of concept, we proposed a virtual optical setup and generated synthetic point spread functions to simulate optical sectioning and hyperspectral imaging in a scattering-prone tissue environment. We used previously acquired confocal microscopy images of sectioned brain organoid for these simulations. The simulation results showed significant suppression of scattering after applying decoding and digital pinholing to the Hadamard pattern-illuminated images. This scattering-free image closely resembles the ground truth, while the simulated wide-field image appeared highly blurred. Furthermore, the reconstructed hyperspectral image accurately reflects spectral information, although its spatial resolution was lower than that of the optically sectioned image. These results demonstrate that optical sectioning and hyperspectral imaging are achievable without light modulation devices.

Although we aimed to simulate conditions as realistically as possible, several factors must be addressed to implement the proposed system in practice. These include data acquisition time, hologram speckle noise, and calibration for accurately determining the positions of digital pinholes and slits. Spectral Hadamard microscopy requires multiple image frames for reconstruction, creating a tradeoff between acquisition time and SNR. In our configuration $\left(n,q\right)$ = (19, 5), a total of 76 frames are required: 19 frames for single Hadamard illumination cycle, repeated 4 times with pattern shifts to suppress the hologram speckle noise through averaging. With a typical exposure time of ∼200 ms for fluorescent imaging, acquiring 76 frames would take ∼15 s. However, since each pixel is illuminated $\left(n+1\right)/2$ times during the sequential illumination of Hadamard pattern – and given *n* = 19 – an effective exposure time of ∼20 ms is sufficient to maintain the SNR, resulting in a reasonable total acquisition time of ∼1.5 s. Modern sCMOS cameras, capable of frame rates 50–500 Hz for 1–16 megapixels (and even several kHz with reduced active pixels), supporting this acquisition time [50]. In implementation, additional frames may be required to address issues such as severe hologram speckle noise or significant scattering in deep tissue imaging. Moreover, enhancing spectral resolution for hyperspectral imaging inherently demands more frames. To maintain reasonable acquisition times with increased frame number, a fast and accurate motorized stage also can be used. Reducing hologram speckle noise could also minimize the number of required frames by eliminating the need for pattern shifts and averaging – in our case, removing the 4 times averaging step. Utilizing partially coherent or incoherent light sources [37], [38], [39] or adopting speckle-free hologram designs [51] could significantly mitigate speckle noise and improve efficiency. Accurate calibration is critical for achieving high-quality digital pinholes and slits. Extremely thin and broadband fluorescent materials are well-suited for precise calibration. Incorrect calibration can result in inaccuracies in hyperspectral data acquisition and substantial signal loss due to misaligned pinhole or slit positions. While this study primarily explored theoretical feasibility through numerical simulations, we believe that this work will advance metasurface-based optical microscopes and that sophisticated metasurfaces could potentially partially replace and enhance conventional optical components [52].

## Supplementary Material

Supplementary Material Details

## References

[1] Butson A. T. (1962). Generalized hadamard matrices. *Proc. Am. Math. Soc.*.

[2] Hedayat A., Wallis W. D. (1978). Hadamard matrices and their applications. *Ann. Stat.*.

[3] Mitrouli M. (2014). Sylvester Hadamard matrices revisited. *Spec. Matrices*.

[4] Zinoviev D., Zinoviev V. (2022). On Sylvester-type constructions of Hadamard matrices and their modifications. ..

[5] Kumari S., Mahato H. (2019). Extension of Paley construction for hadamard matrix. ..

[6] Miyamoto M. (1991). A construction of Hadamard matrices. *J. Comb. Theory Ser. A*.

[7] Yu X., Stantchev R. I., Yang F., Pickwell-MacPherson E. (2020). Super sub-nyquist single-pixel imaging by total variation ascending ordering of the hadamard basis. *Sci. Rep.*.

[8] Zhang Z., Wang X., Zheng G., Zhong J. (2017). Hadamard single-pixel imaging versus Fourier single-pixel imaging. *Opt. Express*.

[9] López-García L., Cruz-Santos W., García-Arellano A., Filio-Aguilar P., Cisneros-Martínez J. A., Ramos-García R. (2022). Efficient ordering of the Hadamard basis for single pixel imaging. *Opt. Express*.

[10] Sun M.-J., Meng L.-T., Edgar M. P., Padgett M. J., Radwell N. (2017). A Russian Dolls ordering of the Hadamard basis for compressive single-pixel imaging. *Sci. Rep.*.

[11] Zhuoran C., Honglin Z., Min J., Gang W., Jingshi S. (2013). An improved Hadamard measurement matrix based on Walsh code for compressive sensing. *2013 9th International Conference on Information, Communications & Signal Processing*.

[12] Zhou Y., Sun Q., Liu Y., Liu J. (2017). Compressed sensing natural imaging via hadamard-diagonal matrix. *2017 4th IAPR Asian Conference on Pattern Recognition (ACPR)*.

[13] Lum D. J., Knarr S. H., Howell J. C. (2015). Fast Hadamard transforms for compressive sensing of joint systems: measurement of a 3.2 million-dimensional bi-photon probability distribution. *Opt. Express*.

[14] Qi Y., Li L., Zhou G., Lim Z. H., Chau F. S., Zhou G. (2020). A single-pixel hyperspectral imager using two-stage Hadamard encoding. *Opt. Commun.*.

[15] Xie H., Lu J., Han J., Zhang Y., Xiong F., Zhao Z. (2023). Fourier coded aperture transform hyperspectral imaging system. *Opt. Lasers Eng.*.

[16] Yi Q., Heng L. Z., Liang L., Guangcan Z., Siong C. F., Guangya Z. (2020). Hadamard transform-based hyperspectral imaging using a single-pixel detector. *Opt. Express*.

[17] Farhi S. L. (2019). Wide-area all-optical neurophysiology in acute brain slices. *J. Neurosci.*.

[18] Parot V. J. (2019). Compressed Hadamard microscopy for high-speed optically sectioned neuronal activity recordings. *J. Phys. D: Appl. Phys.*.

[19] Kim J. (2022). Metasurface holography reaching the highest efficiency limit in the visible via one-step nanoparticle-embedded-resin printing. *Laser Photonics Rev.*.

[20] Zhang Q. (2023). Diffractive optical elements 75 years on: from micro-optics to metasurfaces. *Photonics Insights*.

[21] Shroff H., White H., Betzig E. (2013). Photoactivated localization microscopy (PALM) of adhesion complexes. *Curr. Protoc. Cell Biol.*.

[22] Shroff H., Galbraith C. G., Galbraith J. A., Betzig E. (2008). Live-cell photoactivated localization microscopy of nanoscale adhesion dynamics. *Nat. Methods*.

[23] Hess S. T., Girirajan T. P. K., Mason M. D. (2006). Ultra-high resolution imaging by fluorescence photoactivation localization microscopy. *Biophys. J.*.

[24] Manley S. (2008). High-density mapping of single-molecule trajectories with photoactivated localization microscopy. *Nat. Methods*.

[25] Lelek M. (2021). Single-molecule localization microscopy. *Nat. Rev. Methods Primers*.

[26] Shroff H., White H., Betzig E. (2013). Photoactivated localization microscopy (PALM) of adhesion complexes. *Curr. Protoc. Cell Biol*..

[27] Zhuang X. (2009). Nano-imaging with STORM. *Nat. Photonics*.

[28] Rust M. J., Bates M., Zhuang X. (2006). Sub-diffraction-limit imaging by stochastic optical reconstruction microscopy (STORM). *Nat. Methods*.

[29] Huang B., Wang W., Bates M., Zhuang X. (2008). Three-dimensional super-resolution imaging by stochastic optical reconstruction microscopy. *Science*.

[30] Huang B., Jones S. A., Brandenburg B., Zhuang X. (2008). Whole-cell 3D STORM reveals interactions between cellular structures with nanometer-scale resolution. *Nat. Methods*.

[31] Butler C. (2022). Multi-dimensional spectral single molecule localization microscopy. *Front. Bioinform.*.

[32] Yan R., Moon S., Kenny S. J., Xu K. (2018). Spectrally resolved and functional super-resolution microscopy via ultrahigh-throughput single-molecule spectroscopy. *Acc. Chem. Res.*.

[33] Jeong D., Kim D. (2022). Super-resolution fluorescence microscopy-based single-molecule spectroscopy. *Bull. Korean Chem. Soc.*.

[34] Zhang Z., Kenny S. J., Hauser M., Li W., Xu K. (2015). Ultrahigh-throughput single-molecule spectroscopy and spectrally resolved super-resolution microscopy. *Nat. Methods*.

[35] Titze V. M. (2024). Hyperspectral confocal imaging for high-throughput readout and analysis of bio-integrated microlasers. *Nat. Protoc.*.

[36] Paley R. E. A. C. (1933). On orthogonal matrices. *J. Math. Phys.*.

[37] Deng Y., Chu D. (2017). Coherence properties of different light sources and their effect on the image sharpness and speckle of holographic displays. *Sci. Rep.*.

[38] Lee S., Kim D., Nam S.-W., Lee B., Cho J., Lee B. (2020). Light source optimization for partially coherent holographic displays with consideration of speckle contrast, resolution, and depth of field. *Sci. Rep.*.

[39] Peng Y., Choi S., Kim J., Wetzstein G. (2021). Speckle-free holography with partially coherent light sources and camera-in-the-loop calibration. *Sci. Adv.*.

[40] Kim C., Lee B. (2023). TORCWA: GPU-accelerated Fourier modal method and gradient-based optimization for metasurface design. *Comput. Phys. Commun.*.

[41] Jacques S. L., Wang L., Welch A. J., Van Gemert M. J. C. (1995). Monte Carlo modeling of light transport in tissues. *Optical-Thermal Response of Laser-Irradiated Tissue*.

[42] Wijethilake N., Anandakumar M., Zheng C., So P. T. C., Yildirim M., Wadduwage D. N. (2023). DEEP-squared: deep learning powered De-scattering with Excitation Patterning. *Light: Sci. Appl.*.

[43] Johansson J. D. (2010). Spectroscopic method for determination of the absorption coefficient in brain tissue. *J. Biomed. Opt.*.

[44] Negash A., Mangeat T., Chaumet P. C., Belkebir K., Giovannini H., Sentenac A. (2019). Numerical approach for reducing out-of-focus light in bright-field fluorescence microscopy and superresolution speckle microscopy. *JOSA A*.

[45] Cole R. W., Jinadasa T., Brown C. M. (2011). Measuring and interpreting point spread functions to determine confocal microscope resolution and ensure quality control. *Nat. Protoc.*.

[46] Stallinga S., Rieger B. (2010). Accuracy of the Gaussian point spread function model in 2D localization microscopy. *Opt. Express*.

[47] Gnanasambandam A., Chan S. H. (2022). Exposure-referred signal-to-noise ratio for digital image sensors. *IEEE Trans. Comput. Imaging*.

[48] Huang L., Luo R., Liu X., Hao X. (2022). Spectral imaging with deep learning. *Light: Sci. Appl.*.

[49] Ding K. (2024). Snapshot spectral imaging: from spatial-spectral mapping to metasurface-based imaging. *Nanophotonics*.

[50] Yoon K., Han K., Tadesse K., Mandracchia B., Jia S. (2023). Simultaneous multicolor multifocal scanning microscopy. *ACS Photonics*.

[51] Chen L., Tian S., Zhang H., Cao L., Jin G. (2021). Phase hologram optimization with bandwidth constraint strategy for speckle-free optical reconstruction. *Opt. Express*.

[52] Jo Y., Park H., Yoon H., Kim I. (2024). Advanced biological imaging techniques based on metasurfaces. *Opto-Electron. Adv.*.

