# Supplementary material for: Spectral Hadamard microscopy with metasurface-based patterned illumination
Source: Nanophotonics. 2025 Feb 7;14(8):1171–83. doi: 10.1515/nanoph-2024-0587 (PMC12019938; doi:10.1515/nanoph-2024-0587)
Supplement: Supplementary file 1 — Supplementary Material Details [file j_nanoph-2024-0587_suppl_001.docx]

**Supplementary Information**

Spectral Hadamard microscopy with metasurface-based patterned illumination

Yongjae Jo^1^, Hyemi Park^1, 2^, Seho Lee^1, 2^, and Inki Kim^1, 2, 3, *^

^1^ Department of Biophysics, Institute of Quantum Biophysics, Sungkyunkwan University, Suwon 16419, Republic of Korea

^2^ Department of Intelligent Precision Healthcare Convergence, Sungkyunkwan University, Suwon 16419, Republic of Korea

^3^ Department of MetaBioHealth, Sungkyunkwan University, Suwon 16419, Republic of Korea

*Corresponding author: [inki.kim@skku.edu](mailto:inki.kim@skku.edu)

Yongjae Jo and Hyemi Park contributed equally to this work.

| 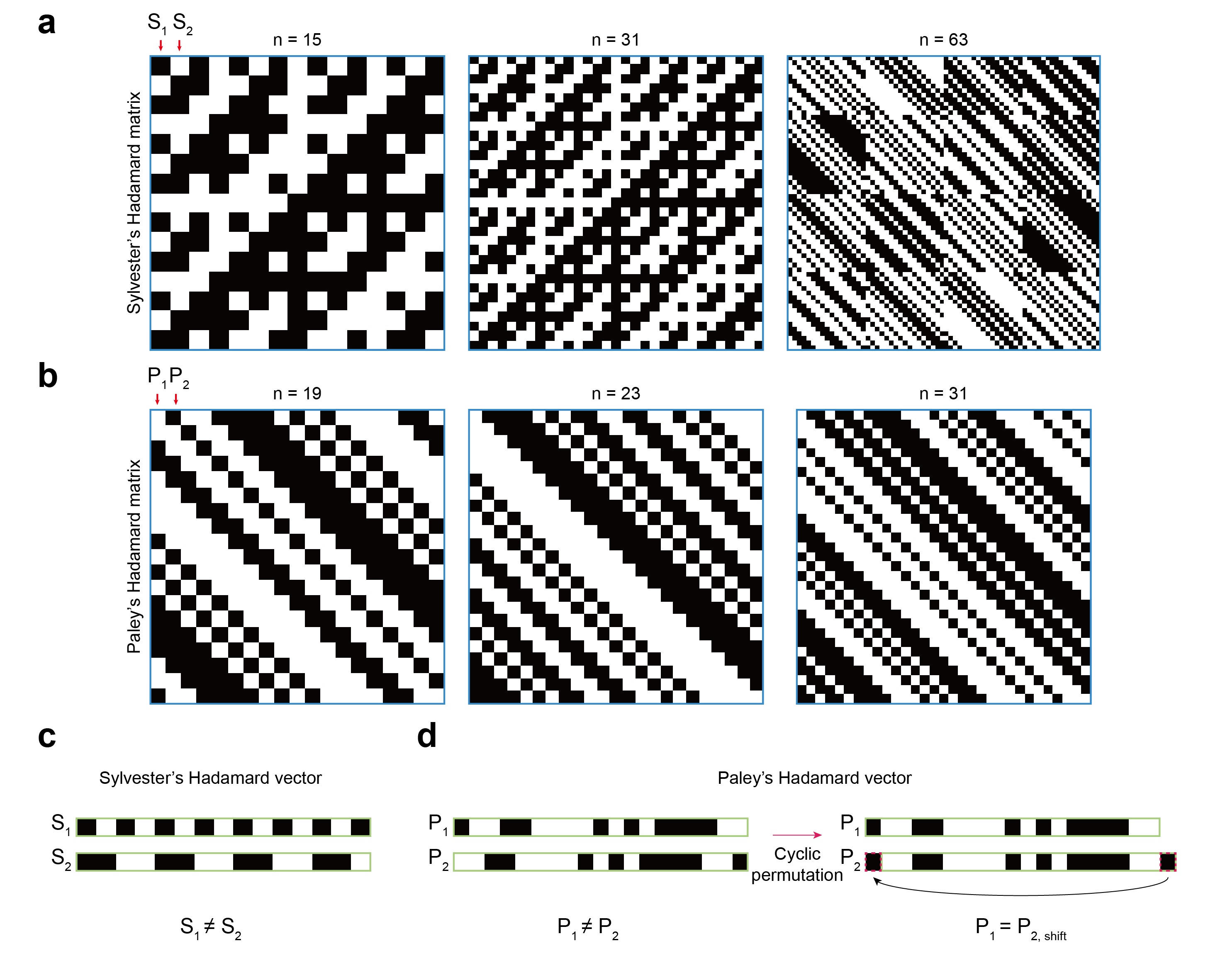 |
| --- |
| **Supplementary Fig. 1 \| Comparison of Sylvester’s and Paley’s Hadamard matrices.** (**a**) Representation of Sylvester’s Hadamard matrices for n = 15, 31 and 63. (**b**) Representation of Paley’s Hadamard matrices using Paley’s construction I for n = 19, 23 and 31. (**c**) Comparison of two column vectors extracted from the Sylvester’s matrix for n = 15, where S1 and S_2_ refer to the labels in (a). (**d**) Comparison of two column vectors extracted from the Paley’s matrix for n = 19, where P1 and P_2_ refer to the labels in (b). While P_1_ is not identical to P_2_, they become identical through cyclic permutation, which relation valid for any pair of vectors extracted from Paley’s matrices. |

| 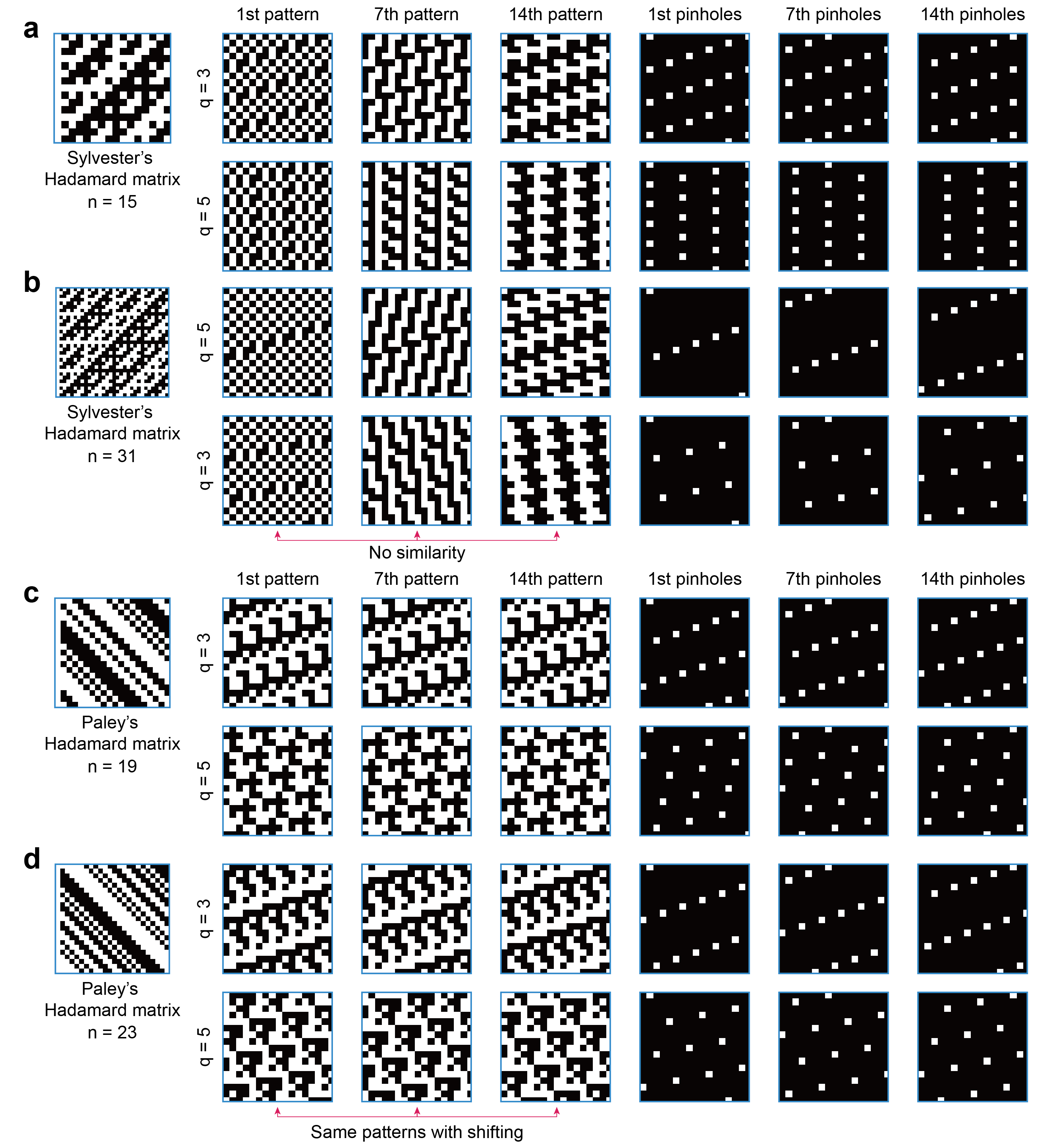 |
| --- |
| **Supplementary Fig. 2 \| Self-similarity of Hadamard patterns generated using Paley’s Hadamard matrix.** (**a, b**) Sylvester’s matrices, and representative Hadamard patterns for illumination and digital pinhole arrangements for (**a**) ($n, q$) = (15, 3) and (15, 5), and (**b**) ($n, q$) = (31, 3) and (31, 5). The Hadamard illumination patterns do not exhibit similarity for a given $n$. (**c, d**) Paley’s matrices, and representative Hadamard patterns for illumination and digital pinhole arrangements for (**a**) ($n, q$) = (19, 3) and (19, 5), and (**b**) ($n, q$) = (23, 3) and (23, 5). Notably, all Hadamard patterns are identical, regardless of the $q$ value or the order of patterns, for a given $n$, a property attributed to the circular matrix structure of Paley’s Hadamard matrices. |

| 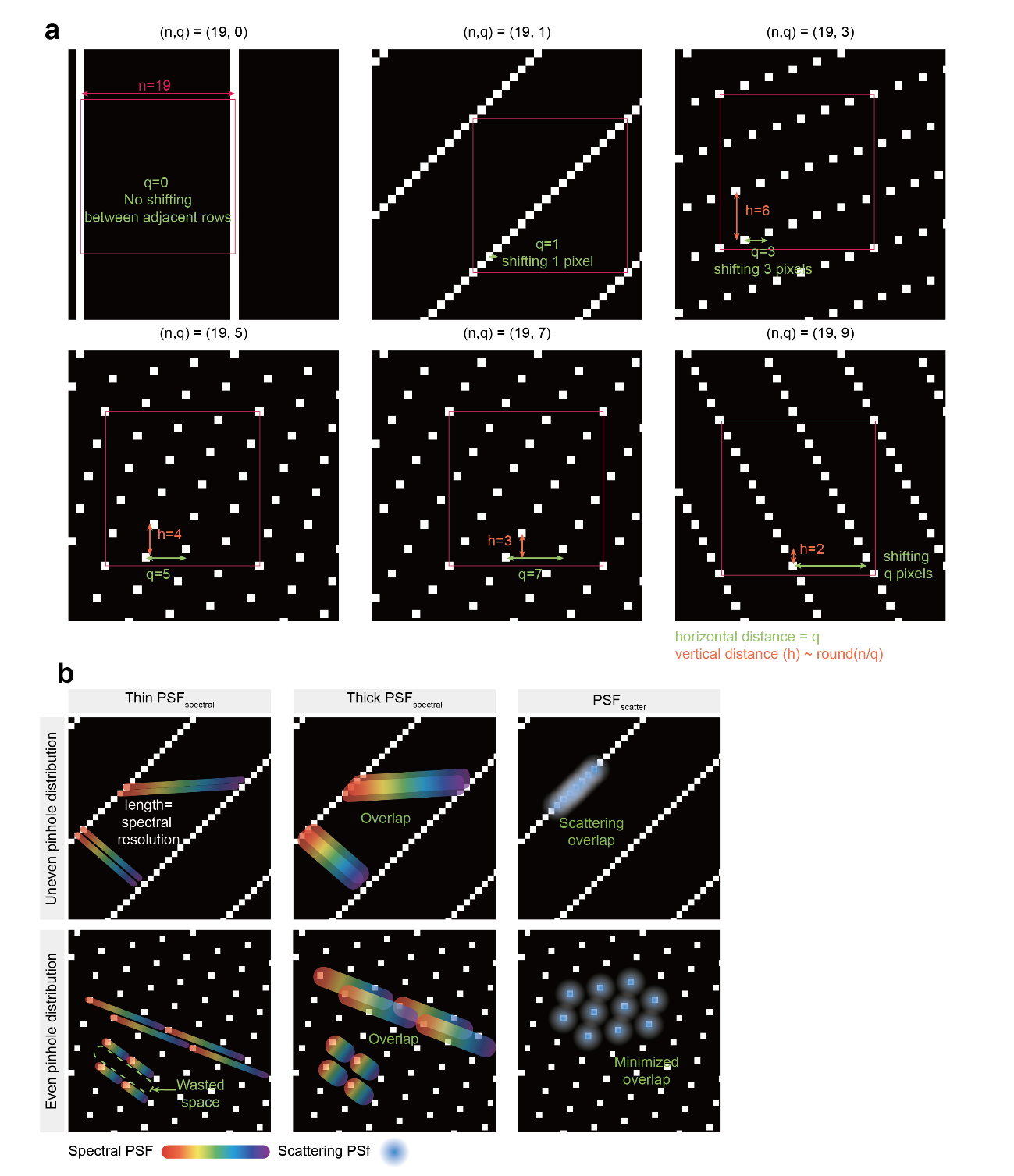 |
| --- |
| **Supplementary Fig. 3 \| Various digital pinhole arrangements with respect to the parameter q.** (**a**) Examples of different digital pinhole arrangements for $n$ = 19 with various $q$ values. The $q$ value determines the horizontal distance between pinholes in adjacent rows, while the vertical distance ($h$) between pinholes in adjacent columns is approximately calculated as $round(\frac{n}{q})$. The magenta square indicates the 19 × 19 pixel of repeating units. (**b**) Effect of digital pinhole arrangements for hyperspectral imaging and optical sectioning. Biased pinhole arrangements are prone to overlapping spectral and scattering signals, whereas evenly distributed pinholes effectively prevent such issues. The dispersion direction can be selected to maximize spectral resolution which is defined as the number of pixels covered by $PSF_{spectral}$. |

| 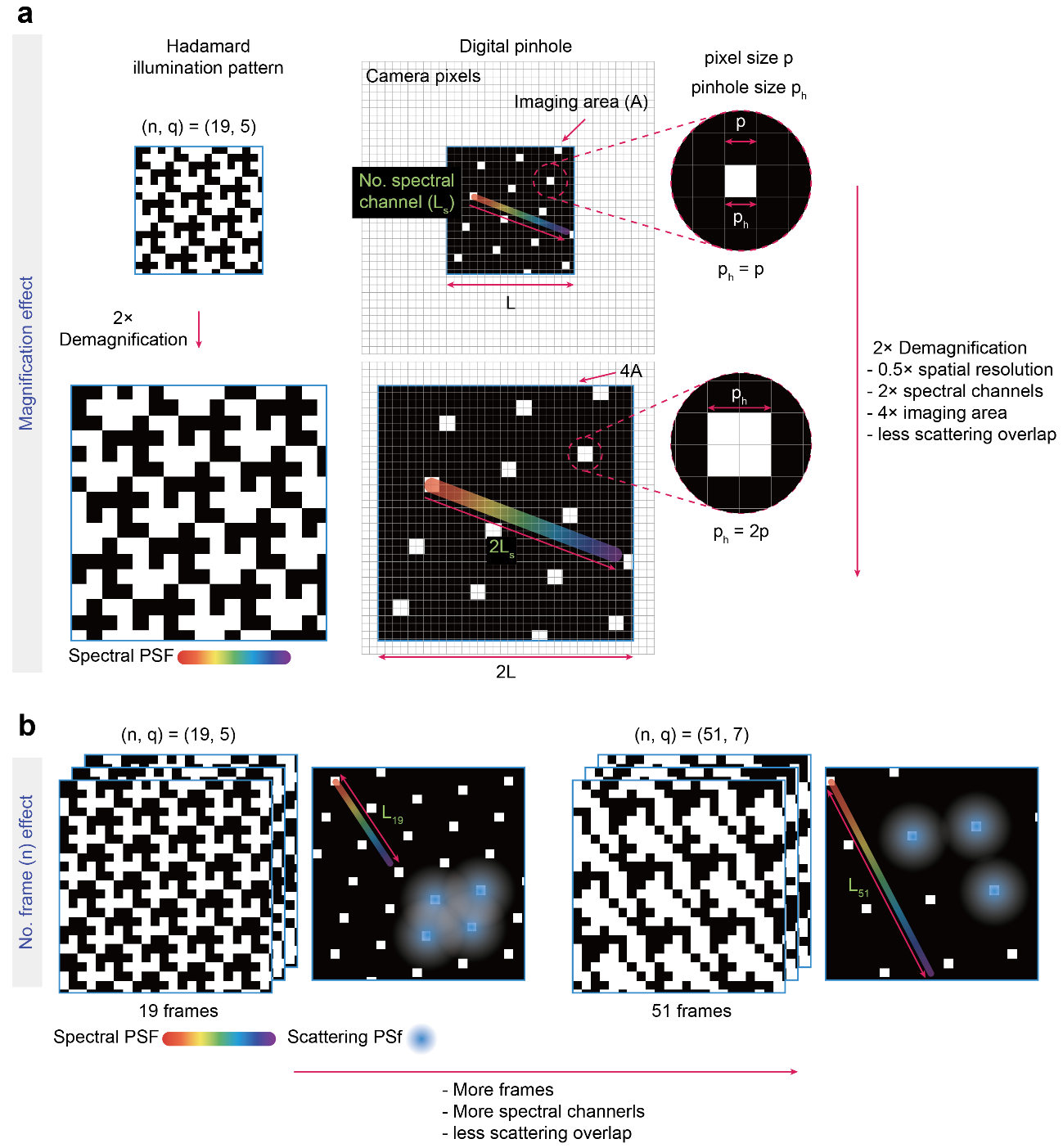 |
| --- |
| **Supplementary Fig. 4 \| Tradeoff between spectral and spatial resolution, number of frames.** (**a**) Pattern magnification affects imaging area, as well as spatial and spectral resolution. For simplicity, we compared scenarios where digital pinhole size (p_h_) matches either a single pixel (p) or double pixels (2p). When the Hadamard pattern is demagnified by a factor of two, the imaging area (A) and spectral resolution (L_s_) increase by a factors of 4 (4A) and 2 (2L_s_), respectively, as the distances between digital pinholes increase. However, this reduces spatial sampling of the hyperspectral image by half, since each digital pinhole—where spectral sampling occurs—covers four pixels. This means that the same spectral information acquired at a digital pinhole is assigned four different pixels, thereby reducing spatial resolution of the hyperspectral image. On the other hand, scattering overlaps are reduced due to the increased inter-pinhole spacing. (**b**) Parameter $n$ influences both the number of spectral channels and scattering overlap. Increasing $n$ requires longer acquisition time because more imaging frames are needed, but it also reduces scattering overlap and enhances spectral resolution by increasing inter-pinhole distances. L_n_ represents the number of pixels available for spectral channels, which increases with the number of frames ($n$). |

| 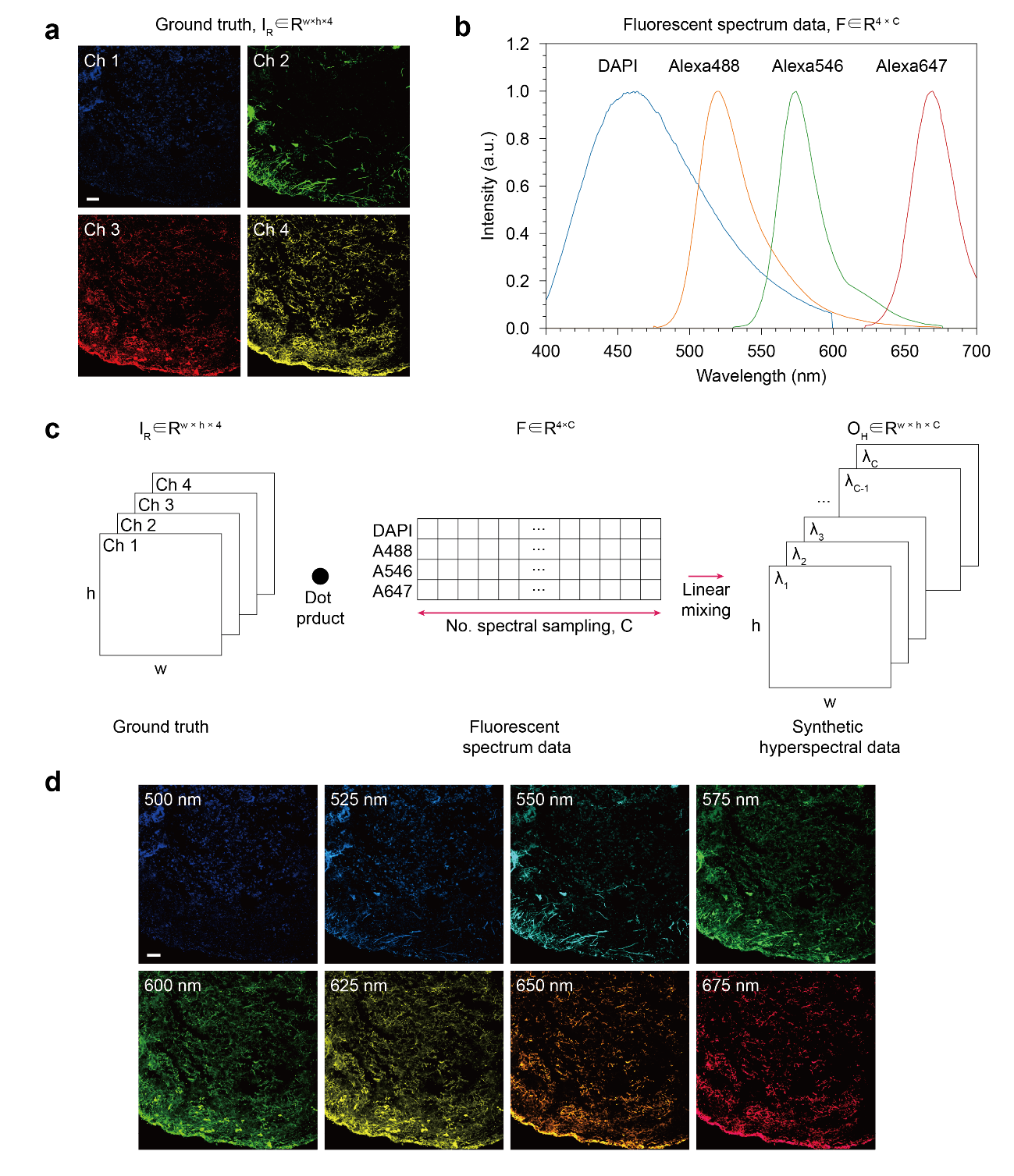 |
| --- |
| **Supplementary Fig. 5 \| Generating synthetic hyperspectral image data from 4-channel ground truth image.** (**a**) 4-channel ground truth image ($I_{R}\in\mathbb{R}^{w\times h\times4}$), and (**b**) fluorescent spectrum data ($F\in\mathbb{R}^{4\times C}$) used for linear mixing. (**c**) Schematic representation of the linear mixing process performed as a dot product between $I_{R}$ and $F$ to generate synthetic hyperspectral image data. (**d**) Representative images selected from the synthetic hyperspectral data. Scale bars: 50 μm (a, b). |
